# Assessment of olfactory recovery after COVID-19: cross-sectional study

**DOI:** 10.1007/s00405-024-08646-5

**Published:** 2024-04-19

**Authors:** Ehab Abou Zaid, Ahmad Mohamed Eltelety, Khaled Omar Azooz, Gouda Ragab, Ahmed Amin Nassar

**Affiliations:** Al Kasr Al Ainy School of Medicine, Cairo University, Cairo, 11562 Egypt

**Keywords:** COVID-19, Olfactory disorders, QOD-NS, Sniffin’ Sticks test, Recovery

## Abstract

**Objective:**

This study aimed to evaluate recovery patterns of olfactory dysfunction among recovered COVID-19 patients, both subjective and objective, and correlate this recovery to the severity of the disease.

**Methods:**

The study recruited 200 patients and assigned them to two equal groups, one of them was a control group. The olfactory function of the study group was assessed via subjective and objective methods at baseline and then monthly for three months, with changes in smell function reported at each visit. These patients underwent chemosensory testing using the Sniffin’ Sticks test and completed the validated Arabic version of the Questionnaire of Olfactory Disorders-Negative Statements (QOD-NS).

**Results:**

Olfactory dysfunction occurred on the first day of COVID-19 symptoms in 37% of participants. Subjective reports suggested smell recovery in 55% after 3 months, but Sniffin' Sticks showed only 1% with normal function, indicating persistent deficits in others. This study revealed smell recovery for 93% of participants (median 14 days), with most (58%) recovering within 2 weeks. No significant links were found between demographics, COVID-smell loss timing, and recovery speed.

**Conclusion:**

Three months after COVID-19, many patients perceive smell recovery, but objective tests reveal shockingly high rates of persistent dysfunction. Further follow-up with objective tests is vital to assess the true burden and potential long-term effects of smell loss.

## Introduction

Olfactory disorders have become more prominent in the patient’s quality of life due to the COVID-19 pandemic, as recent studies show that more than half of COVID-19 patients suffer from olfactory problems [1, 2].

A recent meta-analysis found that while most patients have olfactory disorders that last for a few weeks, around 5% of patients who had olfactory problems at first will still have them after 6 months [3].

The long-term consequences of COVID-19-related smell loss are veiled in uncertainty. Despite extensive research on its early symptoms, the duration and ultimate fate of this olfactory dysfunction remain unclear. This knowledge gap hampers both patients’ understanding of their recovery timeline and healthcare professionals' ability to offer accurate guidance [4, 5].

This study aimed to evaluate recovery patterns of olfactory dysfunction among recovered COVID-19 patients, both subjective and objective, and correlate this recovery to the severity of the disease.

## Materials and methods

### Participants

This cross-sectional study enrolled 100 patients with olfactory dysfunction (OD). Since April 2021, we prospectively examined patients with disordered smell after a SARS-CoV-2 infection at the Otolaryngology department of our tertiary care center from April 2021 to May 2022. Inclusion in the study was restricted to individuals who met the following criteria: they were over 18 years of age, had a confirmed diagnosis of SARS-CoV-2 infection based on a nasopharyngeal PCR test, and presented with olfactory dysfunction (OD) as a manifestation of their illness. The following patients were excluded from the study: (1) < 18 years of age; (2) ongoing COVID-19 symptoms; (3) patients without documented COVID-19 recovery; (4) diagnosed with a neurological condition that would preclude participation, such as dementia; (5) critically ill at time of presentation; (6) negative test result for COVID-19 and no viral symptoms; (7) history of head trauma, allergic rhinitis, chronic rhinosinusitis, previous sinonasal surgery and pre-existing manifestations of olfactory dysfunction; and (8) those who declined to participate in this study.

A group of 100 individuals without COVID-19 history, negative SARS-CoV-2 PCR tests served as the control group. These individuals, including asymptomatic healthcare providers regularly tested with PCR, were evaluated similarly to the intervention group, both subjectively and objectively.

The Research Ethics Committee of the Faculty of Medicine–Cairo University approved this cross-sectional study on 25-4-2021 under the number MD-56-2021.

All patients who participated in the study provided their written consent. All measures of safety for patients, doctors and healthcare workers were applied during patient encounters. Patient education was conducted to explain the type of olfactory tests and their safety. All participants had at least two negative PCR tests before conducting the study.

A thorough clinical history was obtained from all participants. All Patients underwent a full otolaryngology examination, including nasal endoscopy with careful inspection of the olfactory cleft. Basic neurological examinations were done.

Assessment of smell for all patients—both subjectively and objectively—was conducted during the first interview. The assessment was repeated monthly for 3 months. Changes in the olfactory function were reported on each visit.

### Subjective olfactory function assessment

**Fig. 1:**
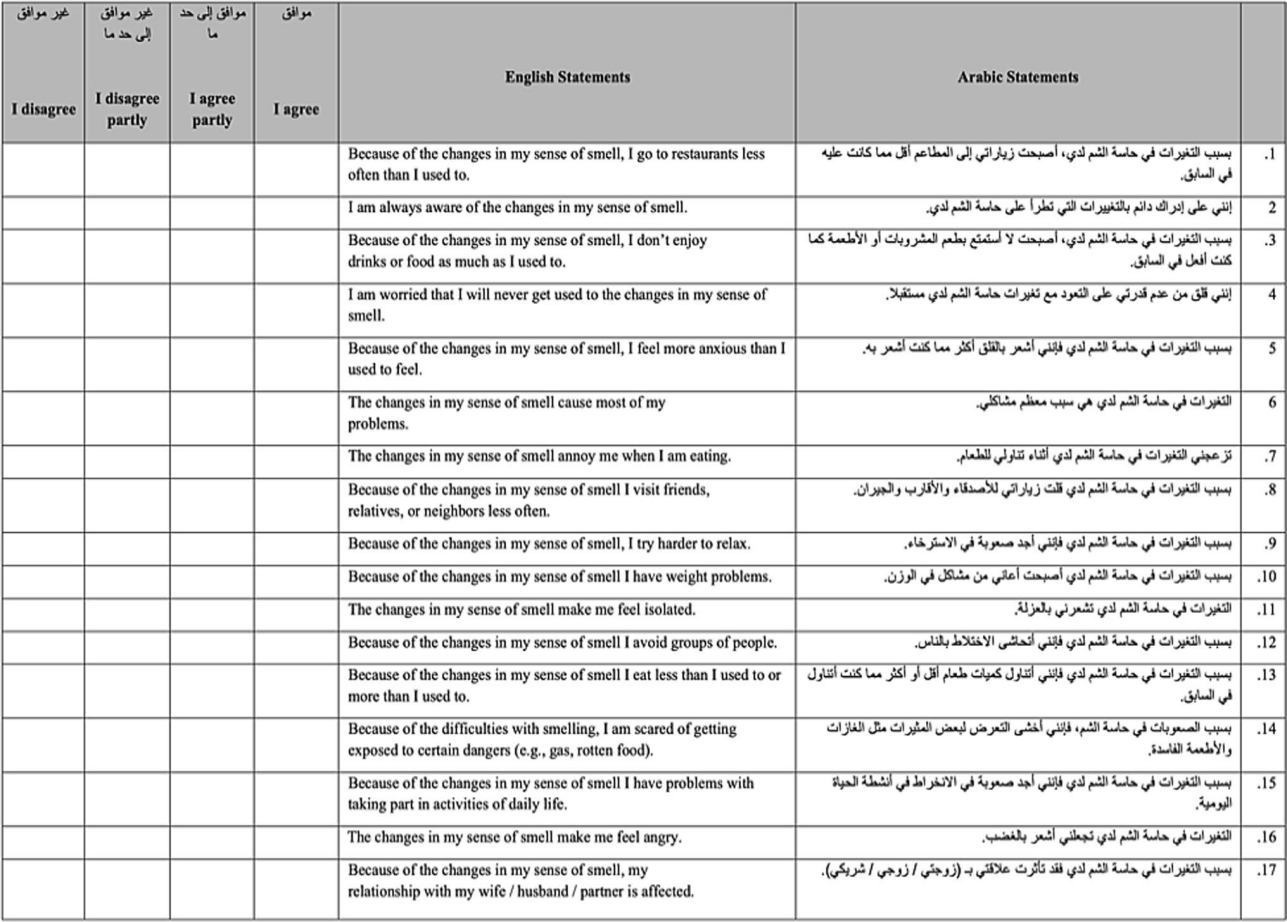
Arabic version of OOD-NS

Patients were asked to rate their olfactory condition before and after COVID-19 infection using the validated Arabic version of the Questionnaire of Olfactory Disorders-Negative Statements (QOD-NS) [6]. The QOD-NS questionnaire is a tool used to assess the degree of olfactory impairment in patients. It consists of 17 negative statements that patients can agree, partly agree, partly disagree, or disagree with, each statement ranging from 0 to 3. A total score of 0 to 51 is calculated, with higher scores indicating a worse olfactory-specific quality of life (Fig. 1).

### Objective olfactory function assessment

Sniffin stick test-12 (SST-12) was validated in 2001 by Hummel et al. This 4-min screening psychophysical test is an odor identification test based on 12 of the 16 odors being sniffed during the identification subdomain part of the original SST [7].

Sixteen smell pens dispensing different odorants were used in the olfactory identification (OI) test. Each pen is presented only once and an interval of at least 30 s is maintained between each presentation to avoid olfactory desensitization. The identification test was scored from 0–16. The test defined a normosmia (SST-12 ≥ 11), hyposmia (10 > SST-12 > 6), or anosmia (SST-12 ≤ 6) based on normative values assessed from more than 1200 patients assessed with SST and olfactive evoked potential for anosmic and hyposmic ones [7].

### Statistical analysis:

Descriptive statistics are presented in the form of mean and standard deviation for normally distributed numerical variables and median with interquartile range for non-normally distributed variables. Numbers and percentages are used for the categorical variables. Kruskal–Wallis and Mann–Whitney U tests were used to compare duration till recovery across sex, age, and duration between acquiring COVID-19 infection and the development of olfactory dysfunction in days. IBM SPSS 26 (SPSS Inc., Chicago, IL, USA). for Windows software was used for the analysis, and a P-value < 0.05 is considered statistically significant.

## Results

The study recruited 200 patients and assigned them to two equal groups. The study group age ranged from 18 to 60 years, with the mean age being 34.92 ± 10.01 years. 65% of the study group participants were female. The mean age of the control group is 32.67 ± 10.01 years. 52% of the control group were female. There was no statistically significant difference between the study and control groups regarding age and gender (*p-value: 0.116, 0.062 respectively*).

Regarding the severity of COVID-19 status among the study population, most of our patients were isolated at home (94%) with mild symptoms with the mean isolation duration being 14.69 ± 2.74 days. The other patients (6%) are isolated at the hospital in the ward setting.

**Fig. 2:**
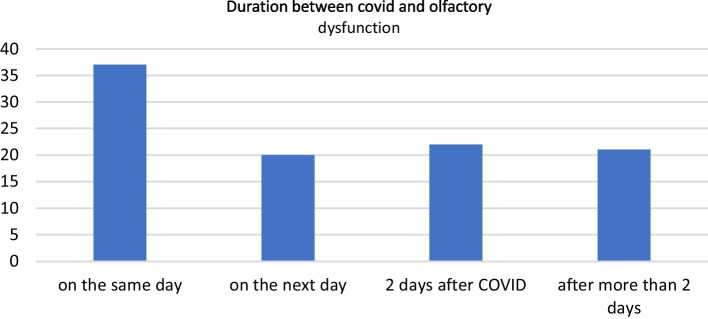
Bar chart for duration between covid 19 onset and onset of olfactory dysfunction

Our cross-sectional study exhibited a significant prevalence of olfactory dysfunction (OD) with sudden onset in most cases and the mean onset was 3.2 days after symptoms of COVID-19 first appeared. According to our subjective evaluation of the study group, we noticed that more than one-third of the study group patients (37%) reported loss of smell on the same day of COVID-19 onset and 20% of the participants were affected on the next day, 22% of the participants suffered from olfactory dysfunction 2 days after COVID-19 and 21% of the participants suffered from olfactory dysfunction after more than 2 days (Fig. 2).

There was a significant recovery of smell function over three months. Complete loss decreased dramatically, while partial loss remained prominent. Importantly, a substantial proportion (55%) regained normal smell (Table 1).

**Table 1:** Subjective characteristics of degree of olfactory dysfunction (more than one symptom may be present)

Degree of olfactory dysfunction	1st visit	2nd visit	3rd visit	4th visit
Complete loss of smell	78	37	8	4
Partial loss of smell	21	59	63	41
Abnormal smell	12	7	7	7
Loss of taste	27	0	0	0
Normal olfactory function	0	0	29	55

In our study regarding the recovery of smell as a subjective symptom, 93% of the participants recovered while only 7% did not recover during the study duration. The median duration till recovery in days was 14 days (IQR = 18).

**Fig. 3:**
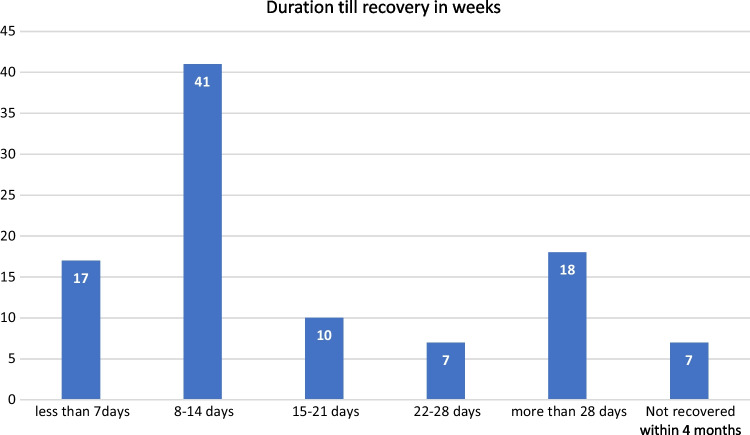
Duration till recovery of smell among study group (subjective assessment)

A substantial proportion of the patients recovered from their smell loss within 2 weeks (58%) and 35% of the patients recovered after the second week (Fig. 3).

In the objective assessment using the Sniffin sticks test, the participants experienced changes in their sense of smell over four visits. Initially, 4% had hyposmia, and 96% had anosmia. Over subsequent visits, there was a gradual improvement, with varying degrees of olfactory dysfunction. After three months, 1% regained normosmia, indicating an overall positive trend in smell function during the study (Table 2).

**Table 2:** Results of objective smell test (Sniffin sticks test) for the study group

Objective smell test	First visit	Second visit	Third visit	Fourth visit
Anosmia	96	77	48	36
Hyposmia	4	23	51	63
Normosmia	0	0	1	1

So, we noted that in our psychophysical testing using the Sniffin stick test after 3 months of follow-up, only one patient regained normal olfactory function and the remaining patients—despite the apparent gradual improvement—still had variable degrees of olfactory dysfunction either anosmia or hyposmia. On the other hand, during subjective analysis, 55% of patients reported normal olfactory function after 3 months duration of follow-up. In some cases, patients subjectively reported normosmia, while chemosensory testing classified them into anosmic or hyposmic category. The category of olfactory dysfunction changing between subjective and chemosensory testing was uncommon. However, it can be stated that the correlation between subjective complaints and chemosensory testing remains controversial.

The study group had a significantly lower olfaction identification (OI) score than the control group with p < 0.001 (3, 13; respectively). while the study group had significantly higher olfactory specific scores (QOD-NS) than the control group with p < 0.001 (17, 0; respectively) (Table 3).

**Table 3:** Comparison of clinical parameters between study and control groups

Characteristics	Study group	Control group	*P* value
OI score, median (IQR)	3 (3–5.75)	13 (10–13)	< 0.001
QOD-NS score median (IQR)	17 (13–24.5)	0 (0–2.00)	< 0.001

Non-parametric tests (Kruskal–Wallis and Mann–Whitney *U* tests) were used to compare the association between duration till recovery and other factors. There is no statistically significant difference between sex, type of isolation, age, duration between COVID and olfactory dysfunction in days and duration till recovery (Table 4).

**Table 4:** Effect of variants on the restoring olfactory function in the study group

	*N*	Median	IQR	*P*-value
Gender				
Male	31	14.0	18	0.535
Female	62	14.0	18	
Type of isolation				
Home	87	14.0	18	0.692
Hospital	6	14.0	12	
Age groups				
Less than 30	27	14.0	18	0.397
30–39 years	36	14.0	16	
40–49 years	20	14.0	11	
50 or more	10	21.0	17	
Duration between covid and olfactory dysfunction (days)				
On the same day	33	14.0	18	0.456
On the next day	19	14.0	14	
2 days after COVID	20	14.0	20	
After more than 2 days	21	14.0	14	

## Discussion

Reports of COVID-19-related OD describe a sudden onset of olfactory impairment, which may be in the presence or absence of other symptoms. However, due to the lack of long-term follow-up, it is unknown what proportion of patients develop persistent post-infectious olfactory dysfunction [8].

Coronaviruses like SARS-CoV-2, are known culprits for post-infectious OD. Nasal epithelial cells show relatively a high concentration of ACE2 receptors used by SARS-CoV-2 to gain entry. The intricate process of olfaction can be disrupted in several ways by viral attacks on the olfactory neuroepithelium. Inflammation, direct damage to smell receptors, and even impaired regeneration of these cells can all contribute to temporary or long-lasting OD [9, 10].

This study aimed to evaluate recovery patterns of olfactory dysfunction among recovered COVID-19 patients, both subjective and objective, and correlate this recovery to the severity of the disease.

In terms of age and gender, the study and control groups were statistically comparable. Thus, the olfactory dysfunction encountered in our study was attributed only to COVID-19 infection.

The present study included 100 participants who experienced OD as a result of a SARS-CoV-2 infection. Similar to previous research findings, the current population has a prominent female predominance (65%). It is noteworthy that this gender disparity was also prevalent in other studies, with female participants' percentages being as follows: 62.5% [11], 54.4% [12], 59% [13] or 68.1% [14] respectively.

Regarding the severity of COVID-19 status among the study population, most of our patients were isolated at home (94%) with mild symptoms. In reported literature by Vaira et al. [16], Lechien et al. [17] and Moein et al. [15], olfactory dysfunction related to COVID-19 was more prevalent in patients with mild disease isolated at home rather than in patients with moderate to severe symptoms isolated at the hospital [15–17]. This is due to higher chemosensory deficits in ambulatory patients. Additionally, as COVID-19 severity increases, non-life-threatening symptoms like chemosensory deficits become less noticeable and less important [18, 19].

Our cross-sectional study exhibited a significant prevalence of OD, and of sudden onset in most cases with the mean onset being 3.2 days. OD occurred on the first day of COVID-19 symptoms in 37% of participants. This result agreed with Speth et al. [5].

Regarding the time of onset of smell dysfunction in patients with COVID-19, it was difficult to figure out a relationship between the findings of the studies reviewed, because of the extent to which they differ from one another. Some of the authors suggest that smell changes may precede the onset of typical disease symptoms like fever, shortness of breath, dry cough, and fatigue [8, 13] These results agreed with our subjective evaluation of our study group as smell loss was the first presenting symptom in some COVID-19 patients.

Furthermore, the Centers for Disease Control and Prevention (CDC) included the loss of smell among the signs and symptoms that can manifest between the second and fourteenth day following exposure to the virus [20].

This study noted that during subjective analysis, 55% of patients reported normal olfactory function after 3 months duration of follow-up. On the other hand, during psychophysical testing using the Sniffin stick test after 3 months of follow-up only one patient regained normal olfactory function and the remaining patients—despite the apparent gradual improvement—still have variable degrees of olfactory dysfunction either anosmia or hyposmia.

This was agreed with the study done by Chiesa-Estomba et al. 2020 stating that 63% of patients reported improvement in their subjective loss of sense of smell after at least 4 weeks. However, the frequency of residual olfactory dysfunction after one month of follow-up was significant, despite the possibility of a later recovery [21].

Another investigation evaluating the resolution of post-COVID-19 olfactory dysfunction at 7 weeks after onset reported that 61.7% of participants self-reported complete recovery, while only 46% were normosmic on psychophysical testing. There is only a moderate correlation between self-reported loss of smell and olfactory testing [22].

Also, studies have shown that patients who self-rated themselves as fully recovered may still have persistent deficits in psychophysical testing [23].

Moreover, several authors have underlined that the subjectivity of self-reporting may lead to underestimation of the prevalence of olfactory dysfunction [15, 19, 24].

The duration of the olfactory dysfunction in COVID-19 disease is still unclear. Initial studies on the duration report a rapid recovery of the symptoms within two weeks, but results were based only on a survey of patients [23].

Our study evaluated the recovery of smell as a subjective symptom. During the study duration, 93% of the participants recovered, while only 7% did not recover. The median duration for recovery was 14 days (IQR = 18). Also, this study stated that 58% of the patients recovered from their loss of smell within two weeks, while 35% of the patients recovered after the second week.

Researchers in the United Kingdom repeated surveys 1 week after the initial survey, revealing that 80% had experienced some recovery, while only 17% remained anosmic. They also noted a “plateau” in recovery after approximately 3 weeks, with a 70% recovery rate for those with anosmia of 3 or more weeks duration [25].

A study published by Lechien et al. described a complete recovery rate of 44% within 5 to 8 days following the resolution of the general symptoms.

In our study, we found an insignificant association between the degree of olfactory recovery and the severity of COVID-19 which indicates that COVID-19 disease is associated with smell recovery irrespective of its degree. However, this result may have occurred because we only assessed patients with mild and moderate disease severities.

It is well known that the subjective perception of olfaction in many people with an olfactory disorder is not congruent with objective measurement data [26].

Based on our results, The incongruence between subjective and objective tests underscores the need for a dual-method approach to accurately assess post-viral olfactory dysfunction. Discrepancies could be explained by qualitative disorders disrupting self-assessment (e.g., parosmia) [13].

Also, the discrepancies in recovery times (from a few days to several months) between individuals could be partly explained by how severely the SARS-CoV-2 virus has damaged the olfactory system [27], even though the mechanisms are still under discussion [17].

In this study, parosmia concerned 12% of patients at the presentation of the disease. Parosmia is experienced in many COVID-19 patients. One study presented the nature of parosmia as the most prevalent as early as 3 months until 1 year after COVID-19 symptoms onset [28].

Parosmia seems to be highly prevalent with mean onset between 8 and 12 weeks after COVID-19, with 10–50% of those reporting initial olfactory dysfunction. Also, it may be a positive predictor of olfactory recovery [29].

Our study used both subjective and objective assessments to evaluate olfactory function. We used the Sniffin’ Sticks test, which is a reliable and validated test for objective smell testing. However, all surveys on olfactory disorders in COVID-19 disease have only used questionnaires so far, and only a few studies have tried to validate the symptomatology using approved olfactory tests [15].

Another strength of this study is that we used the questionnaire on olfactory disorders, which is a validated questionnaire for olfactory assessment. The Questionnaire of Olfactory Disorders (QOD) proved superior to simple “smell questions” from sinus-specific questionnaires like SNOT-22 and Rhinosinusitis Disability Index (RSDI) in distinguishing between patients with normosmia and hyposmia.

The study has limitations, including being cross-sectional, limited to Egyptian patients, and having a small sample size, requiring further validation for larger, longitudinal studies on olfactory dysfunction in COVID-19.

## Conclusion

This study's findings suggest a high prevalence of objective olfactory dysfunction at three months post-COVID-19, necessitating long-term follow-up with psychophysical testing to definitively assess the potential for permanent impairment.

We concluded that there are no statistically significant associations between sex, type of isolation, age, time between COVID-19 diagnosis and olfactory dysfunction onset, and time to olfactory function recovery.

## Data Availability

The data that support the findings of this study are available from the authors upon reasonable request.
